# The Epigenetic *Trans-*Silencing Effect in *Drosophila* Involves Maternally-Transmitted Small RNAs Whose Production Depends on the piRNA Pathway and HP1

**DOI:** 10.1371/journal.pone.0011032

**Published:** 2010-06-14

**Authors:** Anne-Laure Todeschini, Laure Teysset, Valérie Delmarre, Stéphane Ronsseray

**Affiliations:** Laboratoire Biologie du Développement, UMR7622, CNRS-Université Pierre et Marie Curie, Paris, France; Texas A&M University, United States of America

## Abstract

**Background:**

The study of *P* transposable element repression in *Drosophila melanogaster* led to the discovery of the *Trans*-Silencing Effect (TSE), a homology-dependent repression mechanism by which a *P*-transgene inserted in subtelomeric heterochromatin (Telomeric Associated Sequences, “TAS”) has the capacity to repress in *trans*, in the female germline, a homologous *P-lacZ* transgene located in euchromatin. Phenotypic and genetic analysis have shown that TSE exhibits variegation in ovaries, displays a maternal effect as well as epigenetic transmission through meiosis and involves heterochromatin (including HP1) and RNA silencing.

**Principal Findings:**

Here, we show that mutations in *squash* and *zucchini*, which are involved in the piwi-interacting RNA (piRNA) silencing pathway, strongly affect TSE. In addition, we carried out a molecular analysis of TSE and show that silencing is correlated to the accumulation of *lacZ* small RNAs in ovaries. Finally, we show that the production of these small RNAs is sensitive to mutations affecting *squash* and *zucchini*, as well as to the dose of HP1.

**Conclusions and Significance:**

Thus, our results indicate that the TSE represents a *bona fide* piRNA-based repression. In addition, the sensitivity of TSE to HP1 dose suggests that in *Drosophila*, as previously shown in *Schizosaccharomyces pombe*, a RNA silencing pathway can depend on heterochromatin components.

## Introduction

Mobilization of transposable elements (TEs) is regulated by complex mechanisms involving proteins encoded by the TEs themselves, as well as heterochromatin formation and small RNA silencing mechanisms [1]–[11]. Genomic sites containing full-length or defective copies of TEs have been identified which are sufficient to establish complete repression of the other copies of the same family scattered throughout the genome. For example in *Drosophila*, the *flam/COM* locus, located in pericentromeric heterochromatin, represses various families of “Type I” TEs (retrotransposons which transpose *via* an RNA intermediate) [12]–[15] and the TAS (Telomeric Associated Sequence) region of sub-telomeric heterochromatin houses strong regulatory *P* elements (“Type II” TEs whose transposition occurs *via* a DNA intermediate) [2], [16]–[18]. The *flam/COM* locus represses expression of *gypsy*, *Zam*, and *Idefix* in somatic follicle cells, thereby preventing transfer of these retrotransposons to the oocyte [19], [20]. By contrast, *P* element repression by telomeric *P* copies takes place in the germline of both sexes [17], [18], [21], [22] and it is in this tissue that all *P* element transposition steps take place [3], [23], [24]. It has been shown recently that the RNA silencing pathways implicated in both the germline and somatic follicle cells of the ovary rely on the piwi-interacting RNA (piRNAs) silencing pathway [8], although the mechanisms at work in these two tissues differ since some actors of the piRNA machinery are present only in the germline [25]–[28].

The study of the mechanism of *P* element repression in the germline, elicited by telomeric *P* copies, has been facilitated by the use of *P*-transgenes instead of natural *P* transposons. The *P-lacZ* transgene carries an in-frame fusion of the N-terminal region of the transposase with the *E. coli lacZ* gene and can be used as an enhancer-trap [29]. It has been shown that the presence of one or two copies of *P-lacZ* in TAS, can repress another *P-lacZ* copy in *trans*, irrespective of the genomic location of the latter copy [30]–[32]. This repression occurs in the female germline (nurse cells and oocytes), but not in the somatic follicle cells [32]. This phenomenon, termed “*Trans*-Silencing Effect” (TSE) [30], thus allows the precise study of the genetic and phenotypic properties of piRNA-based repression in the context of the germline. It has been shown that TSE displays a maternal effect, epigenetic transmission through meiosis and variegation between egg chambers when repression is incomplete [31], [33]. TSE was also shown to be affected by mutations in genes involved in heterochromatin formation (including HP1) and the piRNA silencing pathway [33]. In particular, TSE was shown to be completely abolished by mutations affecting *aubergine*, *armitage*, *homeless* (*spindle-E*) and a partial dose effect of *piwi* was also found [33]. All these genes have been shown to be necessary for the production of piRNAs in the germline [25].

In the present study, we explore further the genetic and molecular properties of TSE with regard to the piRNA-based mechanism of repression. We first tested the effect on TSE of mutations in *squash* (*squ*) and *zucchini* (*zuc*), encoding two putative nucleases which have been shown recently to be involved in the piRNA pathway [25], [34]. SQUASH and ZUCCHINI both interact with AUBERGINE and mutants exhibit dorso-ventral patterning defects similar to those associated with *aub* mutations. Mutations in *squ* and *zuc* induce the transcription upregulation of *Het-A* and *TART* telomeric retrotransposons and result in the loss of piRNAs in the germline [25], [34]. We first show that the loss of function of *squ* and *zuc* has a very strong negative effect on TSE. Second, we provide the first molecular support of the mechanism of TSE showing that *trans*-silencing is correlated to the presence of *lacZ* small RNAs in ovaries, the levels of these small RNAs being strongly affected by mutations in *squ* and *zuc*. Third, we show that accumulation of these small RNAs in ovaries is also sensitive to a mutation affecting HP1 levels. These results open the possibility of a functional reciprocal dependence between heterochromatin formation and RNA silencing in *Drosophila*. Thus TSE in the fly could parallel the “self-reinforcing loop” of RNA silencing and heterochromatin previously shown to occur in *Schizosaccharomyces pombe* [35]–[37].

## Materials and Methods

### Experimental conditions

All crosses were performed at 25°C and involved 3–5 couples in most of the cases. All ovary *lacZ* expression assays were carried out using X-gal overnight staining as described in Lemaitre *et al*. 1993 [21], except that ovaries were fixed for 6 min [33].

### Transgenes and strains

*P-lacZ* fusion enhancer trap transgenes *(P-1152*, *BQ16*) contain an in-frame translational fusion of the *E. coli lacZ* gene to the second exon of the *P transposase* gene and contain *rosy* ^+^ as a transformation marker [38]. The *P-1152* insertion (FBti0005700) comes from stock #11152 in the Bloomington Stock Center and was mapped at the telomere of the *X* chromosome (site 1A); this stock was previously described to carry a single *P-lacZ* insertion in TAS [30]. However, in our #11152 stock, we have mapped two *P-lacZ* insertions in the same TAS unit and in the same orientation which might have resulted from an unequal recombination event duplicating the *P-lacZ* transgene [33]. *P-1152* is homozygous viable and fertile. *BQ16* is located at 64C in euchromatin of the third chromosome [32] and is homozygous viable and fertile. *P-1152* shows no *lacZ* expression in the ovary, whereas *BQ16* is strongly expressed in the nurse cells and in the oocyte.

Lines carrying transgenes have M genetic backgrounds (devoid of *P* transposable elements), as do the multi-marked balancer stocks used in genetic experiments and the strains carrying mutations used for the candidate gene analysis. The Canton^y^ line was used as a control line, completely devoid of any *P* element or transgene (true “M” line).

### Mutations used for the candidate gene analysis

*Su(var)205*, *squash (squ)* and *zucchini (zuc)* are located on chromosome *2*. Loss of function is lethal in the case of *Su(var)205*, female sterile in the case of s*quash* and z*ucchini*.

*Su(var)2–5* ^05^ (or *Su(var)205* ^05^) was X-ray induced and corresponds to a null allele of *Su(var)205* since it only encodes the first ten amino acids of the HP1 protein [39]. *zuc* and *squ* alleles were isolated from an EMS screen [40]. *zuc^HM27^* contains a stop codon at residue 5, *zuc^SG63^* a substitution of Histidine 169 with a Tyrosine in the conserved HKD domain presumably involved in nuclease activity. *squ^HE47^* and *squ^PP32^* are generated by insertion of stop codons at residues 100 and 111, respectively [34]. Lines carrying mutations of s*quash* and *zucchini* were kindly provided by Attilio Pane and Trudi Schüpbach and the line carrying the *Su(var)2–5* ^05^ allele was kindly provided by Gunter Reuter. All the alleles described above are maintained over a *Cy* balancer chromosome. *Cy* balancer chromosomes have been shown not to affect TSE (unpublished results). Additional information about mutants and stocks are available at flybase: http://flybase.bio.indiana.edu/.

### Quantification of TSE

When TSE is incomplete, variegation is observed since “on/off” *lacZ* expression is seen between egg chambers: that is, egg chambers can show strong expression (dark blue) or no expression, but intermediate expression levels are rarely found. TSE was quantified as previously described [33] by determining the percentage of egg chambers with no expression. We scored the number of these repressed chambers among the first five egg chambers of a given ovariole for ten ovarioles chosen at random per ovary. For a given genotype more than 1000 egg chambers were counted.

### Statistical analysis

The levels of TSE produced in flies of different genotypes were compared using the non-parametric *Mann-Whitney* test, conducted on TSE percentages *per* ovary.

### RNase protection assays (RPA)

Small RNAs from adult flies were extracted using the Ambion mirVana™ miRNA isolation kit. Per each condition, 400 ovaries were used for RNA extraction. Aliquots of 4 µg of small RNAs were used in RPA experiments. The radiolabelled RNA probe homologous to the 5′ region of *P-lacZ* was 150 nt long (position 600 to 750 of the *P{1ArB}* transgene (FBtp0000160)). After purification, probes with a specific activity of 5×10^4^ cpm were used. We used the Ambion mirVana™ miRNA detection kit for RPA experiments. Hybridization was performed overnight at 42°C and digestion of single-stranded RNA was carried out for 45 minutes at 37°C with RNase A/RNase T1. After RNase inactivation, protected fragments were precipitated and separated on a 15% acrylamide/polyacrylamide (19∶1) gel running in 0.5×TBE. Protected fragments were detected by autoradiography after 4 weeks of exposure.

## Results

### Functional assay for the *Trans*-Silencing Effect in *zuc* and *squ* mutants

**Figure 1:**
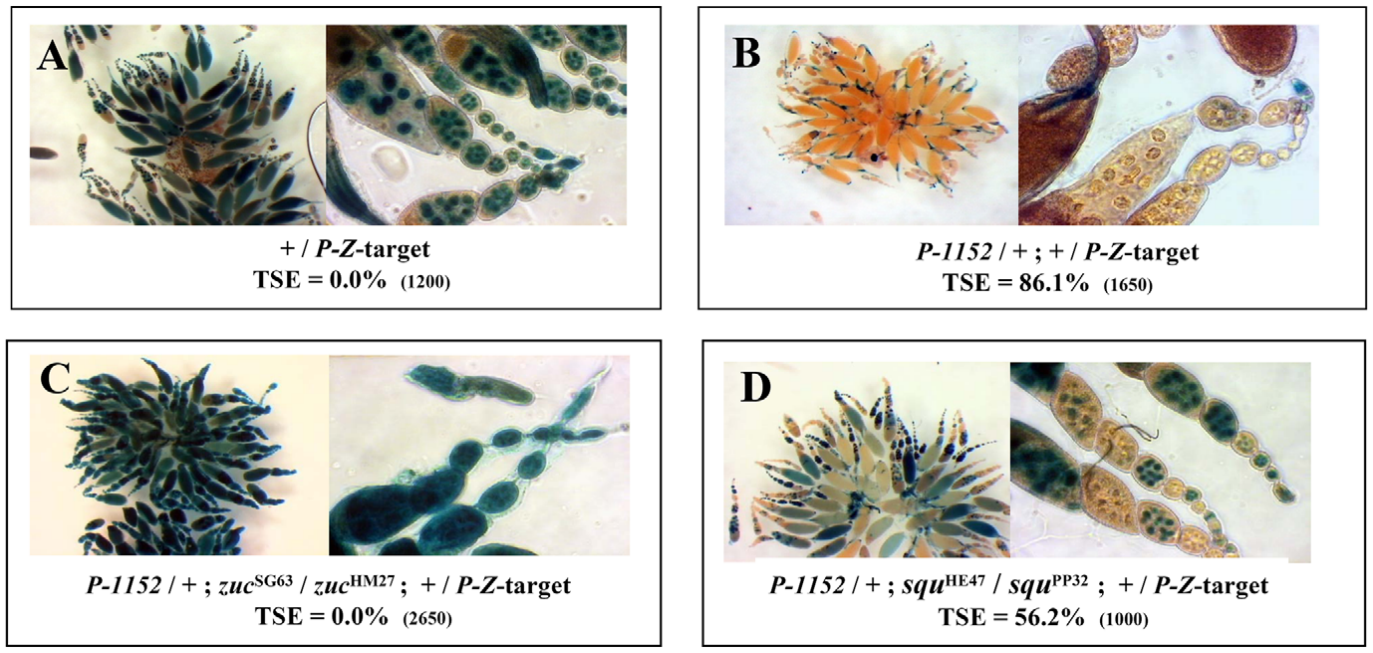
TSE is sensitive to mutations affecting *squash* and *zucchini*. (**A**) Expression control in ovaries of the *P-lacZ* transgene used as a TSE target (*BQ16*, located on chromosome *3*). (**B**) G_1_ females produced from the cross between *P-1152* females and *BQ16* males. (**C–D**) Heteroallelic females for mutant alleles of *zuc* or *squ*: these females have inherited the *BQ16* target paternally and the *P-1152* telomeric silencer from a homozygous *P-1152* female. The maternally-introduced *zuc* or *squ* mutant allele is written first. In each case, the percentage of TSE is given with the total number of egg chambers assayed in parenthesis.

Given the role of *squash* and *zucchini* in the piRNA pathway [25], [34], the effect of mutant alleles of these genes on TSE was tested (Figure 1). For a given assay, a *P-1152* telomeric silencer was combined with a *P-lacZ* target expressed in the female germline, in the absence (TSE positive control), or presence of mutant alleles of the candidate gene. The *P-1152* silencer was inherited, in each case from a homozygous *P-1152* female. The first gene tested was *zucchini* (Figure 1C). The TSE positive control produced a strong repression (Fig. 1B, TSE = 86.1%, n = 1650), whereas females having a heteroallelic *zuc* ^SG63^/*zuc* ^HM27^ genotype showed a complete loss of repression (Fig. 1C, TSE = 0.0%, n = 2650). The same result was found for females having the reciprocally inherited heteroallelic *zuc* ^HM27^/*zuc* ^SG63^ combination (*i.e.* the mutant alleles were inherited by the reciprocal parent: TSE = 0.0%, n = 1600, data not shown). The same analysis was performed for *squash* and the heteroallelic *squ* ^HE47^/*squ* ^PP32^ genotype showed reduced TSE (Fig. 1D, TSE = 56.2%, n = 1000). The reciprocally-inherited heteroallelic genotype, *squ* ^PP32^/*squ* ^HE47^, showed a very similar result (TSE = 56.6%, n = 2200, data not shown). The percentage of TSE observed for each of the two kinds of heteroallelic *squash* mutant females (*squ* ^HE47^/*squ* ^PP32^ and *squ* ^PP32^/*squ* ^HE47^) was compared to that observed for the TSE positive control (Fig. 1B), using the non-parametric *Mann-Whitney* test: in both cases the difference is highly significant (*P<0.001*). By contrast, for both *zuc* and *squ*, no significant effect on TSE was detected for the heterozygous mutants (*zuc*: TSE = 79.4%, n = 1850; *squ*: TSE = 86.8%, n = 2600, data not shown). These levels do not differ from the TSE positive control level (Fig. 1B), as tested with the non-parametric *Mann-Whitney* test. In conclusion, the loss of function of either of these two genes affects TSE, *zuc* having a more severe effect than *squ*, a result consistent with data reported by Pane *et al.* [34] and Malone *et al*. [25] showing that the *zuc* mutant context has a more severe effect than the *squ* mutant context on the production of piRNAs.

### Silencing is correlated to the accumulation of *lacZ* small RNAs in ovaries whose production is sensitive to *squash* and *zucchini* mutations

**Figure 2:**
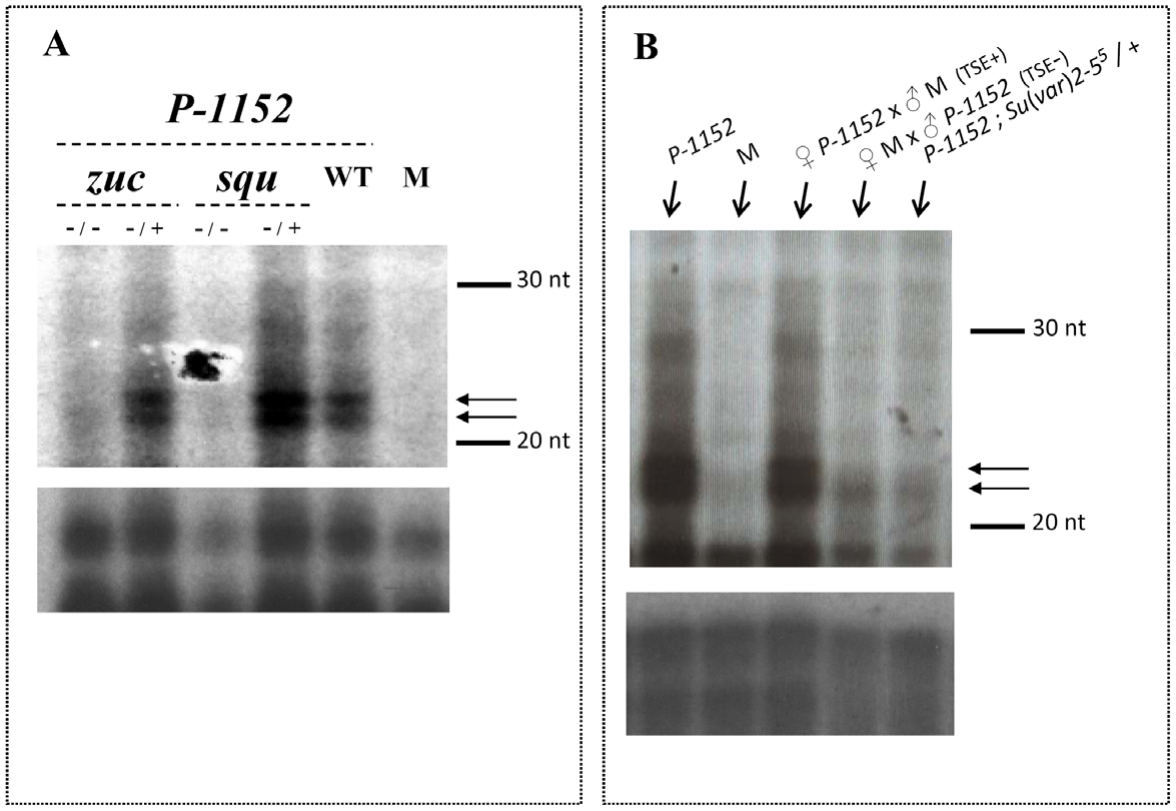
TSE is correlated with the presence of small RNAs whose production depends on the piRNA pathway and HP1. (**A–B**) RNAse protection was carried out using a *lacZ* sense riboprobe (150 nt) hybridized to RNAs extracted from ovaries from 3–6 day-old females. Data concerning the 20–30 nt region are shown together with aspecific bands used as a loading control (shown below). Canton^y^ was used as an M strain, (devoid of any *P* element or *P* transgene). (**A**) Small RNA detection and effect of mutations in *squash* and *zucchini*. WT corresponds to *P-1152* females which are wild-type for both *squ* and *zuc*. Two small RNAs (arrows) are highly abundant in females carrying the *P-1152* telomeric TSE silencer at the homozygous state (WT), but are not detected in ovaries of females devoid of the *P-1152* transgene (M). Females carrying the *P-1152* telomeric silencer at the homozygous state and mutant for *squash* and *zucchini* were analyzed. The same two abundant small RNAs found in *P-1152* (WT) can be detected in females carrying one functional allele of *squ* and *zuc*, but are undetectable in *squ* or *zuc* heteroallelic mutant females. Thus, accumulation of *lacZ* small RNAs occurring in *P-1152* ovaries requires *squ* and *zuc* functions. (**B**) TSE maternal effect and effect of mutations affecting HP1. TSE**+** indicates that this cross allows a strong TSE in G_1_ females due to the maternal transmission of the telomeric *P-1152* silencer, whereas TSE**-** means that only a weak TSE is recovered from this cross in which *P-1152* is inherited paternally. *P-1152* homozygous females and M females were analyzed as positive and negative controls, respectively. The two most abundant small RNAs are indicated by arrows. A strong signal for these small RNAs is obtained for *P-1152* homozygous females and for females having inherited a *P-1152* transgene maternally (TSE+), but is undetectable in negative control M females. The signal for the small RNAs is significantly reduced for females having inherited *P-1152* paternally (TSE-), as well as for *P-1152* homozygous females carrying one null allele of *Su(var)205* which encodes HP1. Therefore, accumulation of *lacZ* small RNAs is correlated to the maternal effect of TSE and depends on HP1 dose.

Since TSE is highly sensitive to mutations in genes involved in the piRNA silencing pathway, we tested whether *lacZ* small RNAs were present in ovaries of females which carry the *P-1152* telomeric silencer locus and, if so, whether the production of these small RNAs requires the *squ* and *zuc* functions. We used an RNAse protection assay to detect *lacZ* small RNAs in ovaries from females carrying two copies of *P-1152* and otherwise wild-type, heterozygous or heteroallelic mutants for *squ* and *zuc*. Ovaries from the M line Canton^y^ were also analyzed as a negative control. RNAse protection analysis allowed detection of two abundant small RNAs in ovaries from homozygous *P-1152* females (Fig. 2A, lane 5 and Fig. 2B, lane 1 – arrows to the right of the autoradiography), which were not detected in M females (Fig. 2A, lane 6 and Fig. 2B, lane 2). Females heterozygous for *squ* or *zuc* mutant alleles, also exhibited abundant accumulation of the *lacZ* small RNAs of the same size in ovaries (Fig. 2A, lanes 2 and 4, respectively). By contrast, these RNAs were almost undetectable for females heteroallelic for mutant alleles of *squ* or *zuc* (Fig. 2A, lanes 1 and 3, respectively). This analysis shows that telomeric *P-lacZ* silencer transgenes produce *lacZ* small RNAs in the ovary and that loss of function of *squ* and *zuc* has a strong negative effect on the accumulation of these *lacZ* small RNAs. In addition, as for the TSE assay, no dose effects for *squ* or *zuc* were observed on *lacZ* small RNAs accumulation.

### Accumulation of *lacZ* small RNAs in ovaries is correlated with the maternal effect of TSE

TSE was shown to exhibit a maternal effect: crossing females carrying a telomeric transgene with males carrying a target transgene produces G_1_ females which show strong TSE, whereas the reciprocal cross produces G_1_ females showing only weak TSE [31]–[33]. TSE also shows maternal inheritance since this maternal effect presents a remanence which can extend through six generations following the reciprocal G_0_ crosses [33]. TSE is therefore, at least in part, epigenetically transmitted through meiosis. TSE maternal inheritance can also be observed in the presence of a telomeric silencer alone, *i.e*. in the absence of the target transgene [32]. We thus tested if *lacZ* small RNA detection in ovaries parallels the maternal effect of TSE. RNAse protection analysis allowed detection of the two abundant small RNAs in G_1_ females produced by the two (*P-1152* x M) reciprocal crosses, but the intensity of the signal obtained with the progeny of the (female *P-1152* x male M) cross (cross TSE+) was higher than that of the progeny of the reciprocal cross which induces only a weak level of TSE (cross TSE-) (Fig. 2B, lanes 3 and 4). This difference becomes particularly clear, when a comparison is made between the signal intensities of the aspecific bands shown below the *lacZ* small RNAs. However, the signal from TSE- females is not null, a result which is consistent with the weak but non null level of TSE (around 10%) which can be induced in this cross [32], [33]. In conclusion, the presence of *lacZ* small RNAs in ovaries is detected in a manner which is correlated to the maternal effect of TSE.

### Accumulation of *lacZ* small RNAs is sensitive to HP1 dose

TSE was shown previously to be sensitive not only to mutations in genes involved in the piRNA pathway, but also to mutations in genes involved in heterochromatin formation, such as *Su(var)205* which encodes HP1 [33]. For *Su(var)205*, a particularly clear dose effect on TSE was observed. We thus tested if the presence of *lacZ* small RNAs in ovaries is affected in *P-1152* females having only one dose of the *Su(var)205* gene compared to wild-type. RNAse protection was performed as previously on females carrying two copies of *P-1152* and heterozygous for *Su(var)2–5* ^05^, an amorphic allele of *Su(var)205*. Figure 2B (lane 5) shows that the level of small RNAs detected for females having two copies of *P-1152* and only one dose of *Su(var)205* is strongly reduced when compared to *P-1152* wild-type females (Fig. 2B, lane 1). Indeed, with one dose of HP1, the level of these small RNAs is comparable to that of females carrying a single paternally-inherited *P-1152* copy (Fig. 2B, lane 4). Under these two latter conditions, comparable low levels of TSE were also found [33]. Therefore, the effect of mutations affecting HP1 on TSE [33], as for *squ* and *zuc* mutations, can be correlated to a significant reduction in the accumulation of small RNAs in ovaries produced by the telomeric *P-1152* silencer locus.

## Discussion

### *Trans*-Silencing Effect, a typical piRNA germline repression mechanism

*Trans*-silencing was previously shown to be strongly impaired by mutations affecting several components of the piRNA silencing pathway (AUBERGINE, ARMITAGE, HOMELESS, PIWI) [8], [33]. By contrast, TSE was not impaired by mutations affecting R2D2, a component of the siRNA pathway [33], [41], or LOQUACIOUS, a component of both the miRNA and endo-siRNA pathways [33], [42]–[44]. This indicates that TSE likely involves the piRNA silencing pathway, a hypothesis which is consistent with the fact that TSE is restricted to the germline [32], the tissue in which the “canonical” piRNA pathway functions [25], [26]. Further, SQUASH and ZUCCHINI were found to interact with AUBERGINE and to localize to the nuage, a cytoplasmic organelle surrounding the nurse cell nuclei, which also contains AUBERGINE and ARMITAGE and appears to be involved in RNA silencing [34]. *squ* and *zuc* mutations were also shown to affect piRNA production in ovaries at the cytological 42AB repetitive sequence cluster, a typical piRNA-producing genomic region [25]. Regarding TE repression in the germline, *squ* and *zuc* mutants were found to derepress transcription of the telomeric retrotransposons *Het-A* and *TART* [34] and of the *I* factor, a retrotransposon involved in a *Drosophila* system of hybrid dysgenesis [45], [46]. It is noteworthy that the *I* factor and the *Het-A* retrotransposons have also been found to be sensitive to *aub*, *armi* and *hls* (*spn-E*) [5], [45], [47]. The genetic analysis reported here shows that TSE is also highly sensitive to *zuc* and *squ* mutations (Figure 1). TSE is therefore sensitive to mutations affecting all the genes of the germline piRNA pathway tested and thus appears to represents a *bona fide* piRNA-based repression.

The presence of *lacZ* small RNAs in ovaries of females carrying a TSE silencer was therefore investigated using RNase protection analysis. In addition, paternal *vs* maternal transmission of the telomeric silencer was compared. Indeed, TSE was previously shown to have a maternal effect, *i.e.* strong repression occurs only when the telomeric silencer is maternally inherited, whereas a paternally-inherited telomeric silencer has weak or null repression capacities [32], [33], [48]. More precisely, it was shown genetically that TSE requires inheritance of two components, a maternal cytoplasmic component plus a chromosomal copy of the transgene, but these two components can be transmitted separately [33]. Indeed, a paternally-inherited telomeric transgene can be “potentiated” by a maternally-inherited cytoplasm from a female bearing a silencer. This interaction also functions between telomeric silencers located on different chromosomal arms [32]. The RNase protection analysis reported here shows that: 1- *P-1152,* a telomeric *P-lacZ* silencer produces small *lacZ* RNAs in ovaries (Figure 2A–B); 2- *P-1152 lacZ* small RNA accumulation is negatively affected in *squ* and *zuc* mutants (Figure 2A); 3- maternal transmission of *P-1152* leads to accumulation of higher levels of these small RNAs than that observed upon paternal *P-1152* transmission (Figure 2B). We have reproduced these results with independent RNAse protection assays (two experiments for the effect of each mutant and three experiments for the maternal effect). The size of the small RNAs detected here appears smaller (around 22–23 nt) than that corresponding to piRNAs as characterized by deep sequencing (23–28 nt, [8]), but they are consistent with piRNAs as detected by RNAse protection assays in other studies [49]: this can result from the RNAse protection protocol which tends to reduce the size of the RNAs detected. In conclusion, our results strongly suggest that the *lacZ* small RNAs in *P-1152* oocytes may correspond to cytoplasmically-transmitted piRNAs mediating the maternal effect of TSE, as well potentiating a paternally-inherited telomeric silencer [33].

### Towards a mutual dependence between RNA silencing and heterochromatin formation

TSE was previously shown to be sensitive to mutations affecting HP1 since a negative, dose-dependent, effect on TSE was found with two loss of function alleles of *Su(var)205* (including *Su(var)2–5* ^05^) [33]. RNase protection analysis shows here that *lacZ* small RNA accumulation is also negatively affected by the dose of HP1 (Figure 2B). Although we cannot exclude that this effect may be indirect, this opens the possibility that some piRNA-producing loci depend on the presence of HP1 itself at the locus to produce piRNAs. A similar model was recently proposed for *rhino*, a HP1 homolog, mutations of which strongly reduce the production of piRNAs by dual strand piRNA- producing loci [28]. The authors propose that *rhino* is required for the production of the long precursor RNAs which are further processed to produce primary piRNAs. Note that in their study, *rhino* mutants were shown to have a drastic effect on the production of piRNAs by the *X*-chromosome TAS locus [28]. A similar situation may therefore exist for HP1 at this locus and, if so, it would be interesting to characterize more precisely the function of HP1 in the production of piRNAs at the TAS locus.

HP1 was shown to be present at TAS [50], [51]. A first possibility would be that HP1 stimulates transcription of the TAS locus as a classical transcription factor, independent of any heterochromatic role at this locus. Consistent with this, it was shown that PIWI, a partner of HP1 [52], promotes euchromatin histone modification and piRNA transcription at the third chromosome TAS [49]. The precise status of TAS, however, remains complex since some studies have shown that TAS exhibit some of the properties attributed to heterochromatin [53]–[55] and carry primarily heterochromatic histone tags [56]. Therefore, a second possibility would be that HP1 enhances the heterochromatic status of TAS in the germline, such that production of aberrant transcripts being processed into piRNAs is enhanced. This would result in a “heterochromatin-dependent RNA silencing pathway”. Examples of heterochromatin formation that depends on RNA silencing (“RNA-dependent heterochromatin formation”) have been described in numerous species including yeast [37], ciliates [57] and plants [58]. In *Drosophila*, this type of interaction has been described for variegation of pigment production in the eye linked to the insertion of the *white* gene in different types of heterochromatin structures [59], [60], as well as for heterochromatin formation at telomeres in the germline [51]. Therefore, telomeric regions in fly may be submitted to both RNA-dependent heterochromatin formation [47], [51] and heterochromatin-dependent RNA silencing. RNA silencing may favor heterochromatin formation that in turn potentiates RNA silencing, resulting in a functional positive loop between transcriptional gene silencing and post-transcriptional gene silencing. In such cases, RNA silencing and heterochromatin may not only reinforce each other but may also be functionally interdependent. Such bidirectional reinforcement between RNA silencing and heterochromatin formation was demonstrated in *S. pombe* since: 1- deletion of genes involved in RNA silencing were shown to derepress transcriptional silencing from centromeric heterochromatic repeats and was accompanied by loss of Histone 3 Lysine 9 methylation and *Swi6* (a HP1 homolog) delocalization [37]; 2 - *Swi6* was found to be required for the propagation and the maintenance of the RNA Induced Transcriptional Silencing (RITS) complex at the *mat* locus, a complex involved in amplification of RNA silencing [35], [61]. A positive loop between RNA silencing and heterochromatin formation may therefore also be at play in the *Drosophila* germline. According to this model, the epigenetic transmission of TSE through meiosis, (*i.e*. six generations of maternal transmission of the silencer are required to elicit a strong TSE following maternal inheritance of a cytoplasm devoid of *lacZ* piRNAs [33]) would underlie progressive establishment of this loop. Note that RNAi-dependent DNA methylation in *Arabidopsis thaliana* was shown to occur progressively over several consecutive generations [62].

Since TSE can be considered as a sub-phenomenon within *P* regulation, it may underlie epigenetic transmission of the *P* element repression. *P* element mobilization is responsible for a syndrome of germline abnormalities, known as the “P-M” system of hybrid dysgenesis which includes a high mutation rate, chromosomal rearrangements, male recombination and an agametic temperature-sensitive sterility called GD sterility (Gonadal Dysgenesis) [63]. *P-*induced hybrid dysgenesis is repressed by a maternally inherited cellular state called the“ P cytotype” [3], [23], [64], [65]. The absence of *P*-repression is called M cytotype. G_1_ females produced from the cross (P cytotype females × M cytotype males) present a strong capacity for repression, whereas females produced from the reciprocal cross present a weak capacity for repression [64]. In the subsequent generations, cytotype is progressively determined by the chromosomal *P* elements but the influence of the initial maternal inheritance can be detected for up to five generations [64], [66]. Therefore, P cytotype exhibits partial epigenetic transmission through meiosis. Furthermore, the identification and use of telomeric *P* elements as P cytotype determinants [2], [16]–[18], has made it possible to show that P cytotype (like TSE) involves a strictly-maternally inherited component (called the pre-P cytotype) [67], is sensitive to mutations affecting HP1 [2], [68] and *aubergine* [1], [69] and is correlated to maternal deposition of piRNAs [70]. Some of these properties are also found for the *I* factor which is responsible for the occurrence of another system of hybrid dysgenesis (“I-R” system) [45], [46], [71]–. TSE therefore parallels germline regulation of TEs (*P*, *I*), and does not resemble regulation of TEs in the somatic follicle cells (*gypsy*, *ZAM*, *Idefix* [19], [20]) for which no epigenetic transmission of repression capacities through meiosis has been described so far. It will be interesting to test if previously described cases of RNA-dependent heterochromatin formation show the reciprocal dependence, thus being able to form a positive loop.
